# FAPI radiopharmaceuticals in nuclear oncology and theranostics of solid tumours: are we nearer to surrounding the hallmarks of cancer?

**DOI:** 10.1007/s12149-025-02022-x

**Published:** 2025-03-11

**Authors:** Irene García Megías, Ludmila Santiago Almeida, Adriana K. Calapaquí Terán, Kim M. Pabst, Ken Herrmann, Francesco Giammarile, Roberto C. Delgado Bolton

**Affiliations:** 1 Department of Diagnostic Imaging (Radiology) and Nuclear Medicine, University Hospital San Pedro and Centre for Biomedical Research of La Rioja (CIBIR), Logroño, La Rioja Spain; 2 Department of Nuclear Medicine, University Hospital of Toledo, Toledo, Spain; 3 Division of Nuclear Medicine, Department of Anesthesiology, Oncology and Radiology, Faculty of Medical Sciences, Campinas University, Campinas, Brazil; 4 Servicio Cántabro de Salud, Santander, España; 5 Department of Pathology, University Hospital “Marqués de Valdecilla”, Santander, Spain; 6 Instituto de Investigación Sanitaria Valdecilla, IDIVAL, Santander, Spain; 7 Department of Nuclear Medicine, West German Cancer Center, University Hospital Essen, University Duisburg-Essen, Essen, Germany; 8 German Cancer Consortium (DKTK), Partner Site University Hospital Essen, Essen, Germany; 9 Nuclear Medicine and Diagnostic Imaging Section, Division of Human Health, Department of Nuclear Sciences and Applications, International Atomic Energy Agency (IAEA), Vienna, Austria

**Keywords:** Cancer, FAPI, Nuclear oncology, Theranostic, PET/CT, Radiopharmaceutical therapy

## Abstract

[^18^F]FDG PET/CT is the most widely used PET radiopharmaceutical in oncology, but it is not exempt of diagnostic limitations. FAPI have emerged as a great tool in the management of several different solid tumours in which [^18^F]FDG is not able to provide enough information. The aim of this work was to evaluate the available evidence on diagnostic and therapeutic applications of PET/CT with FAPI radiopharmaceuticals. We underwent a non-systematic review focusing in the utility of FAPI radiopharmaceuticals in PET/CT diagnosis and in the treatment of several malignancies. FAPI radiopharmaceuticals present characteristics that can potentially overcome some known diagnostic limitations of [^18^F]FDG. FAPI radiopharmaceuticals present a high target-to-background ratio (TBR) in many solid tumours such as oesophageal cancer, gastric cancer, pancreatic cancer, hepatic cancer, colorectal cancer, breast cancer, ovarian, cervical cancer, and head and neck cancer. Available evidence suggests the high TBR improves sensitivity and specificity compared to [^18^F]FDG, especially for the detection of lymphadenopathies and peritoneal metastases, and may improve patient management and radiation treatment planning. Moreover, it is important to underline the potential theranostic application of FAPI radiopharmaceuticals.

## Introduction

Since the 1990s, [^18^F]FDG has been the most widely used radiopharmaceutical for PET/CT in oncology [1, 2]. Nowadays, the interest in precision and personalised medicine has led to the search for new radiopharmaceuticals with the potential of improving patient management. New radiopharmaceuticals should present higher sensitivity and specificity than [^18^F]FDG and also permit theranostic applications [3]. The limitations of [^18^F]FDG are well-known, including high physiological uptake in certain structures, increased uptake in inflammation or infection, and low or lack of uptake by several tumours [1–4]. FAPI-X (referring with “FAPI-X” to a generic FAPI chemical compound) radiopharmaceuticals seem to overcome some of these limitations.

The tumour mass is made up of tumour cells and tumoral stroma, the latter accounting for up to 90% of the tumour mass. The stroma or tumoral microenvironment is composed of different cellular subtypes among which there are cancer-associated fibroblasts (CAF). Fibroblast activation protein (FAP), a type II cell membrane–bound serine peptidase, is over-expressed in the surface of CAF and is the target of FAP inhibitors (FAPI) used in PET/CT imaging [5–7]. A noteworthy fact is that FAP is over-expressed in a high percentage of epithelial tumours, making FAPI-based radiopharmaceuticals pan-tumour markers as has been demonstrated [8, 9]. FAP is also related to a faster tumoral growth, increased proliferation and angiogenesis, and is associated with a worse prognosis, conferring FAPI radiopharmaceuticals a prognostic value [10].

One of the advantages of FAPI-X radiopharmaceuticals is that the PET/CT procedure is more simple, as it does not require any special preparation in contrast with [^18^F]FDG PET/CT, especially regarding fasting and blood glucose monitoring, which are not necessary. Furthermore, FAP is almost non-present in normal tissue and is rapidly eliminated through the urinary tract, making the background activity very low. These conditions offer a high target to background ratio (TBR). Nevertheless, PET/CT with FAPI-X radiopharmaceuticals PET/CT is not exempt of false positives (FP), as some benign processes can show FAPI-X uptake, such as healing and fibrotic processes, muscular tissue, as well as the endometrium and mammary parenchyma in premenopausal women [11]. Overall, the biodistribution of FAPI-X radiopharmaceuticals, usually presenting a low expression in healthy tissues, is also a favourable aspect when considering targeting FAPI for therapeutic purposes, as is the case of theranostic applications (Table 1). The aim of this review is to summarize the available evidence on FAPI-X radiopharmaceuticals in solid neoplastic processes for PET/CT and potential therapeutic applications.

**Table 1:** Comparison of the advantages and disadvantages between FAPI-X PET/CT and [^18^F]FDG PET/CT

Criteria	Radiopharmaceutical	
	FAPI-X	[^18^F]FDG
Availability	Lower	Wide
Volume of evidence	Lower	Higher
Physiological uptake	Lower	Higher
Pitfalls	Healing and fibrotic processes, muscle tissue, endometrium and breast	Inflammation and infection
Therapy	Allows theranostic	Does not allow therapy

## Material and methods

This is a non-systematic review of articles focusing on FAPI-X radiopharmaceuticals used for PET/CT imaging in the management of tumours in which [^18^F]FDG PET/CT shows limitations. The management of these neoplastic processes includes diagnosis, response evaluation and follow-up. We also focused on the promising theranostic applications of FAPI-X with different radionuclides, such as [^177^Lu], [^225^Ac], and [^90^Y].

**Fig. 1:**
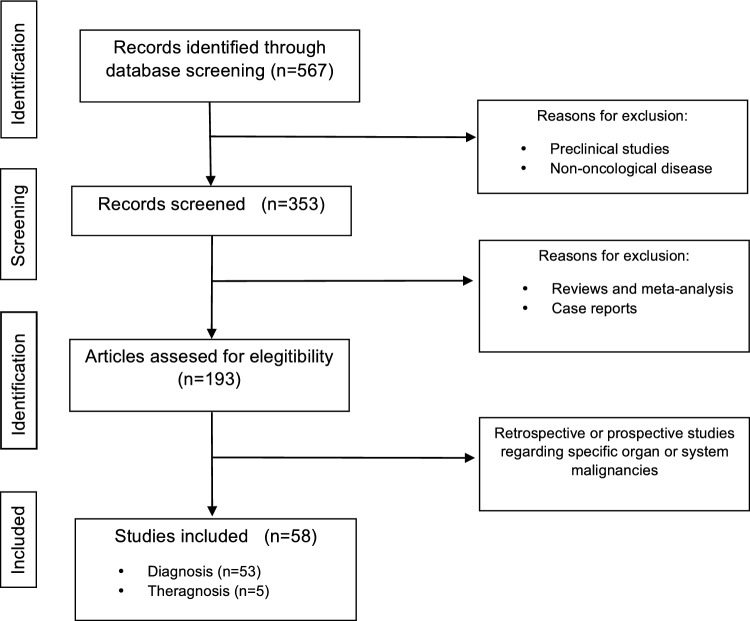
Flow chart for study identification

The inclusion criteria were studies with FAPI-X radiopharmaceuticals marked with either [^18^F] or [^68^Ga], without a minimum number of patients. The exclusion criteria were reviews, full articles not available in English and case reports. Figure 1 presents the flow chart for study identification.

Regarding nomenclature, whenever the chemical compound is specified in the original article it is indicated here, but “FAPI-X” is used to refer to a generic FAPI chemical compound.

## Results

### Digestive tract tumors

#### Oesophageal cancer

[^18^F]FDG PET/CT is an appropriate tool in the staging of oesophageal cancer as part of the multidisciplinary management [12]. FAPI-X radiopharmaceuticals have demonstrated a better detection of primary tumours, including those with a small volume, nodal involvement and distant metastasis (Table 2). Liu et al. reported higher SUVmax with [^68^Ga]Ga-DOTA-FAPI-04 PET/CT in primary lesions (13.8 vs. 10.9 ± 6.8; *p* = 0.004), a better specificity of nodal involvement (2 FP vs. 29 FP uptake) and a better detection of metastatic lesions (25/25 lesions detected by [^68^Ga]Ga-DOTA-FAPI-04 PET/CT) [13]. Wegen et al. reported similar results with [^68^Ga]Ga-FAPI-46 PET/CT, with good accuracy in detecting primary lesions, secondary lymph nodes and distant disease, when compared to [^18^F]FDG PET/CT [14].

**Table 2:** Comparison between FAPI-X PET/CT and [^18^F]FDG PET/CT evidence for each clinical application in digestive malignancies

	Digestive malignancies		
Tumour	Application	FAPI-X	[^18^F]FDG
Oesophageal Cancer	Uptake	Higher TBR [15] and SUVmax [13]	Lower TBR [15] and SUVmax [13]
	T	Good accuracy and better delineation including those with a small volume [13, 14]	Difficult for delineation including those with a small volume [13]
	N	Less FP and better specificity [13, 14]	Weaker for local lymph node evaluation with more FP [13, 14]
	M	Better detection [13, 14]	Weaker detection [13, 14]; Strong evidence of efficacy in locally advanced tumours [12]
	Radiation planning	Higher TBR and better delineation of GTV prior to radiation treatment [15, 16]	*N/A*
	Prognostic	GTV associated with PFS and OS [17]	*N/A*
Gastric Cancer	Uptake	Higher TBR [20] Better in non-intestinal-type [7, 12] and GIST [6, 7, 27] Limited in early stages	Can underestimate lesions due to higher background uptake in normal gastric mucosa Lower TBR [6, 7] Lower GIST detection rate (53.8% vs. 80, 2%, *p* < 0.001) [27] Limited in early stages
	T	Higher detectability (90.3%) [18, 19] Limitations in early gastric cancer evaluation (37.5%, p > 0.05) [18]	Lower detectability (77.4%, *p* = 0.008) [18] Limitations in early gastric cancer evaluation (25.0%, p > 0.05) [18]
	M	Useful for detecting peritoneal dissemination (91.7%) [18, 24, 25] Global sensitivity of 94.44% [19]	Peritoneal metastases 41.7%, *p* = 0.031, [18] Global sensitivity of 61.11% [19]
	Response to therapy	Early changes in % SUVmax and %TBR can predict the therapeutic response to neoadjuvant [26]	Less predictive in early stages [26]
Pancreatic Cancer	Uptake	Helpful in differentiating benign from malignant lesions [29] SUVmax increases with FAP expression [31]	Difficult to discriminate between primary tumours and inflammatory pathology [2, 7] Can identify unknown primary tumours in the pancreas [28]
	T	Higher sensitivity (100%) [30]	Lower sensitivity (73.1%) [30]
	N	Higher SUVmax (8.6) and sensitivity (81.8%) [30]	Lower SUVmax (2.7) and sensitivity (59.1%) [30]
	M	Higher SUVmax (7.9) and sensitivity (91.5%) [30]	Lower SUVmax (3.5) and sensitivity (44.0%). [30]
	Prognostic	Information on the aggressiveness of the tumour providing prognostic information [33]	*N/A*
	Therapy	Preclinical studies: Useful in RT planification [35]; applicable to future RPT	*N/A*
Hepatocellular carcinoma	T	Higher avidity in primary tumours (SUVmax = 8.4; TBR = 13.2), more intra-hepatic lesions (sensitivity and specificity of 100%) [36–38] Useful in the evaluation of hepatic nodules [36]	Limited in well-differentiated hepatocellular carcinoma, high baseline liver activity (SUVmax = 4.2; TBR = 9.5) [36]
	N	Higher detectability [38]	Lower detectability [38]
	M	Better detection of peritoneal metastases [38]	Worse detection [38]
Colorectal Cancer	T	Higher specificity (100.0%) [45]	Lower specificity (85.3%) [45]
	N	Higher specificity (100.0%) [45]	Relevant role ranging from staging to detection of recurrent disease [2, 43, 44] Lower specificity (81.8%) [45]
	M	Higher sensitivity for peritoneal metastases (100%) [45]	Lower sensitivity for peritoneal metastases (55%) [45]

FAPI-X: FAPI-X PET/CT; [^18^F]FDG: [^18^F]FDG PET/CT; SUVmax: standardized uptake value; TBR: tumour to background ratio; FP: false positive; FN: false negative; N/A: not applicable; MRI: magnetic resonance imaging; GTV: gross tumour volume; GIST gastrointestinal stromal tumours; Uptake: Degree of Radiopharmaceutical Uptake; T: tumour; N: nodes; M: metastases; RT: radiotherapy; RPT: radiopharmaceutical therapy

The detection or lack of detection of nodal disease and metastatic lesions changes patient management, which is crucial when planning the most adequate therapy. The higher TBR with [^68^Ga]Ga-FAPI-46 allows better delineation of tumour volumes when planning radiotherapy compared to CT and allows calculating additional boost to metastatic nodes [15]. Compared to [^18^F]FDG, [^68^Ga]Ga-FAPI-X also improves the precision of the delineation of the gross tumour volume (GTV) prior to radiation treatment [16].

The prognostic value of FAPI-Xs PET/CT has been also studied. A retrospective study in 45 patients demonstrated that GTV in [^68^Ga]Ga-FAPI-04 was associated with progression-free survival (PFS) and overall survival (OS) [17].

#### Gastric cancer

Gastric cancer is usually diagnosed in advanced stages due to its insidious symptoms. [^18^F]FDG PET/CT can underestimate the extent of the disease in some cancer subtypes, in particular in the non-intestinal-type [7, 12]. In this scenario, FAPI-X PET/CT could have an important role [6, 7]. The main problem when staging gastric tumours using [^18^F]FDG PET/CT is its low sensitivity to detect primary lesions due to the high background uptake in the normal gastric mucosa, conditioning a low TBR. Also, detecting pathological lymph nodes and peritoneal dissemination is limited due to the low [^18^F]FDG uptake or low TBR.

Miao et al. compared [^68^Ga]FAPI-04 PET/CT with [^18^F]FDG PET/CT in gastric cancer patients showing that [^68^Ga]FAPI-04 was better in the initial evaluation with higher detectability of primary lesions (90.3% vs. 77.4%; *p* = 0.008) and peritoneal metastases (91.7% vs. 41.7%; *p* = 0.031), although both radiopharmaceuticals showed limitations in early gastric cancer evaluation (37.5% vs. 25.0%; *p* > 0.05) [18]. Another study comparing both radiopharmaceuticals also showed higher sensitivity for [^68^Ga]FAPI-04 PET/CT compared to [^18^F]FDG PET/CT, with 94.44% versus 61.11%, respectively [19]. One of the main advantages of [^68^Ga]FAPI-04 PET/CT is the higher contrast with the low background, which provides a higher certainty when reporting active tumoral cells [20]. FAPI-X radiopharmaceuticals can also be used in PET/MRI systems in this indication [21–23]. As already mentioned, different studies have shown that FAPI-X PET/CT can also be useful for the detection of peritoneal metastases, a finding that completely changes the management of the disease by avoiding unnecessary surgeries [24, 25].

Different parameters derived from FAPI-X PET/CT studies can predict the response to treatment (Table 2). In this situation, [^68^Ga]FAPI-04 PET/CT has demonstrated to outperform [^18^F]FDG PET/CT, as early changes in %SUVmax and %TBR can predict the therapeutic response to neoadjuvant chemotherapy [26].

FAPI-X radiopharmaceuticals can also be interesting in the management of gastric cancer types that typically present low [^18^F]FDG, such as gastrointestinal stromal tumours (GIST), with a study reporting a better detection rate for [^18^F]FAPI-42 PET/CT versus [^18^F]FDG PET/CT, with 80.2% versus 53.8% (*p* < 0.001), respectively [6, 7, 27].

#### Pancreatic cancer

Pancreatic cancer is an aggressive tumour and its diagnosis is often late leading to high mortality rates. [^18^F]FDG PET/CT presents some limitations in the management of pancreatic cancer, mainly related to the identification of primary tumour and the uptake seen in inflammatory pathology [2, 7]. In some patients presenting as unknown primary tumours, [^18^F]FDG PET/CT locates the primary tumour in the pancreas [28].

PET/CT with FAPI-X radiopharmaceuticals can help differentiate benign from malignant lesions [29]. Also, compared to [^18^F]FDG PET/CT, FAPI-X PET/CT presents higher sensitivity in the evaluation of primary lesions, secondary lymph nodes and distant metastases, resulting in more precise staging (Table 2). Pang et al. compared [^68^Ga]Ga-FAPI-X PET/CT to [^18^F]FDG PET/CT showing a higher uptake and sensitivity in the primary lesion (SUVmax 21.4 vs. 4.8; sensitivity 100% vs. 73.1%), nodal involvement (SUVmax 8.6 vs. 2.7; sensitivity 81.8% vs. 59.1%) and distant metastases (SUVmax 7.9 vs. 3.5; sensitivity 91.5% vs. 44.0%). Furthermore, in this study [^68^Ga]Ga-FAPI-PET/CT upstaged 6/23 patients [30]. An observational study of 64 patients concluded that SUVmax increases with tumour FAP expression and was a more accurate tool when compared to CT and [^18^F]FDG PET/CT [31]. As seen in initial evaluation, FAPI-X PET/CT tends also to change the staging of patients with recurrent disease [32]. Overall, FAPI-X PET/CT shows a high rate of upgrading staging in both primary and recurrent disease. FAPI-X PET/CT can also provide information on the aggressiveness of the tumour providing prognostic information [33].

PET/MRI with FAPI-X radiopharmaceuticals could be interesting in the management of pancreatic cancer [21]. A study in 33 patients demonstrated that multisequence MRI helps in the assessment of lesions that could not be properly evaluated due to inflammatory FAPI-X uptake, especially the following MRI sequences: T1-weighted-imaging (WI), T2-WI, diffusion-WI, and apparent diffusion coefficient or ADC). They also concluded it could be helpful as a one-stop-shop for diagnosing small liver metastases, recommending for this specific indication the sequence contrast-enhanced T1-WI [34].

The high specificity and the high TBR for FAPI-X PET/CT in pancreatic tumours have two interesting applications, one is in the scenario of radiation therapy planification [35], and the other is in the use of FAPI-X as targets for radiopharmaceutical therapy (RPT), although for the latter the results available are mostly preclinical.

#### Hepatocellular carcinoma and other primary liver tumours

In patients with well-differentiated hepatocellular carcinoma (HCC), [^18^F]FDG PET/CT usually shows low [^18^F]FDG uptake by the tumour, the degree of uptake usually not being significantly different from the surrounding non-tumoral hepatic tissue baseline activity. Normal baseline liver activity is usually higher than in other regions of the body, such as the mediastinal blood pool. Therefore, low-level uptake lesions are difficult to interpret in [^18^F]FDG PET and previously unknown lesions may be masked hindering the detectability of these lesions with [^18^F]FDG. In contrast, a potential role of [^18^F]FDG PET/CT could be to characterise these lesions, confirming the absence of high-grade or undifferentiated hepatic lesions that would present high [^18^F]FDG uptake.

FAPI-X PET/CT has been studied in the evaluation of hepatic nodules among patients with suspicion of hepatic carcinoma. A study in 17 patients concluded that [^68^Ga]FAPI-04 was an adequate radiopharmaceutical in this scenario, as it showed a high sensitivity for detecting malignant lesions including poorly differentiated subtypes (mean SUVmax 8.36 ± 4.21 and mean TBR 13.15 ± 9.48) [36]. Another study in 20 patients compared [^18^F]FDG PET/CT to [^68^Ga]FAPI-04 PET/CT in primary hepatic tumours. They concluded that primary lesions showed higher avidity in the FAPI-X study (sensitivity and specificity of 100%) [37]. A head-to-head comparison investigated the role of [^18^F]FAPI-X PET/CT in the initial staging of HCC versus [^18^F]FDG PET/CT with promising results. [^18^F]FAPI-X PET/CT detected more intra-hepatic lesions as well as lymph nodes and peritoneal metastases. These findings conditioned an upgrade in 12/67 patients (upgrade in T staging) with changes in their therapeutic management [38].

#### Biliary tract tumours

Biliary tract tumours are rare and aggressive neoplasms that arise from the gallbladder, from the cystic duct, or from the biliary tree [39]. [^18^F]FDG PET/CT shows limitations in the management of these entities. A prospective study of 18 patients showed a potential role of [^68^Ga]FAPI-X PET/CT in the staging of biliary tract neoplasms when compared to [^18^F]FDG [40].

Cholangiocarcinoma is the most common biliary tract cancer and usually presents a poor prognosis with high mortality rates. The implementation of FAPI-X PET/CT could have an interesting role in the management of these patients. Zhang et al. studied a cohort of 44 patients with suspected cholangiocarcinoma that underwent [^68^Ga]FAPI-04 PET/CT and [^18^F]FDG PET/CT within 1 week, including 30 patients who underwent simultaneous abdominal [^68^Ga]FAPI-04 PET/MRI. The findings were confirmed by histopathology or radiographic follow-up. They demonstrated better detection rates of the primary tumour, of the nodal involvement and of the peritoneal lesions with [^68^Ga]FAPI-04 PET/CT. On the other hand, [^18^F]FDG demonstrated better results regarding liver and bone metastases. Moreover, in the 30 patients that underwent multisequence [^68^Ga]FAPI-04 PET/MRI, they found the MRI component was helpful in delimitating the primary lesions and detecting hepatic metastases compared to PET/CT [41].

The role of FAPI-X radiopharmaceuticals has also been investigated in the delineation of the tumoral volume in radiotherapy planning in biliary tract cancer, among other neoplastic entities, having found changes in the MTV when using FAPI-X PET/CT compared to [^18^F]FDG PET/CT [42].

**Fig. 2:**
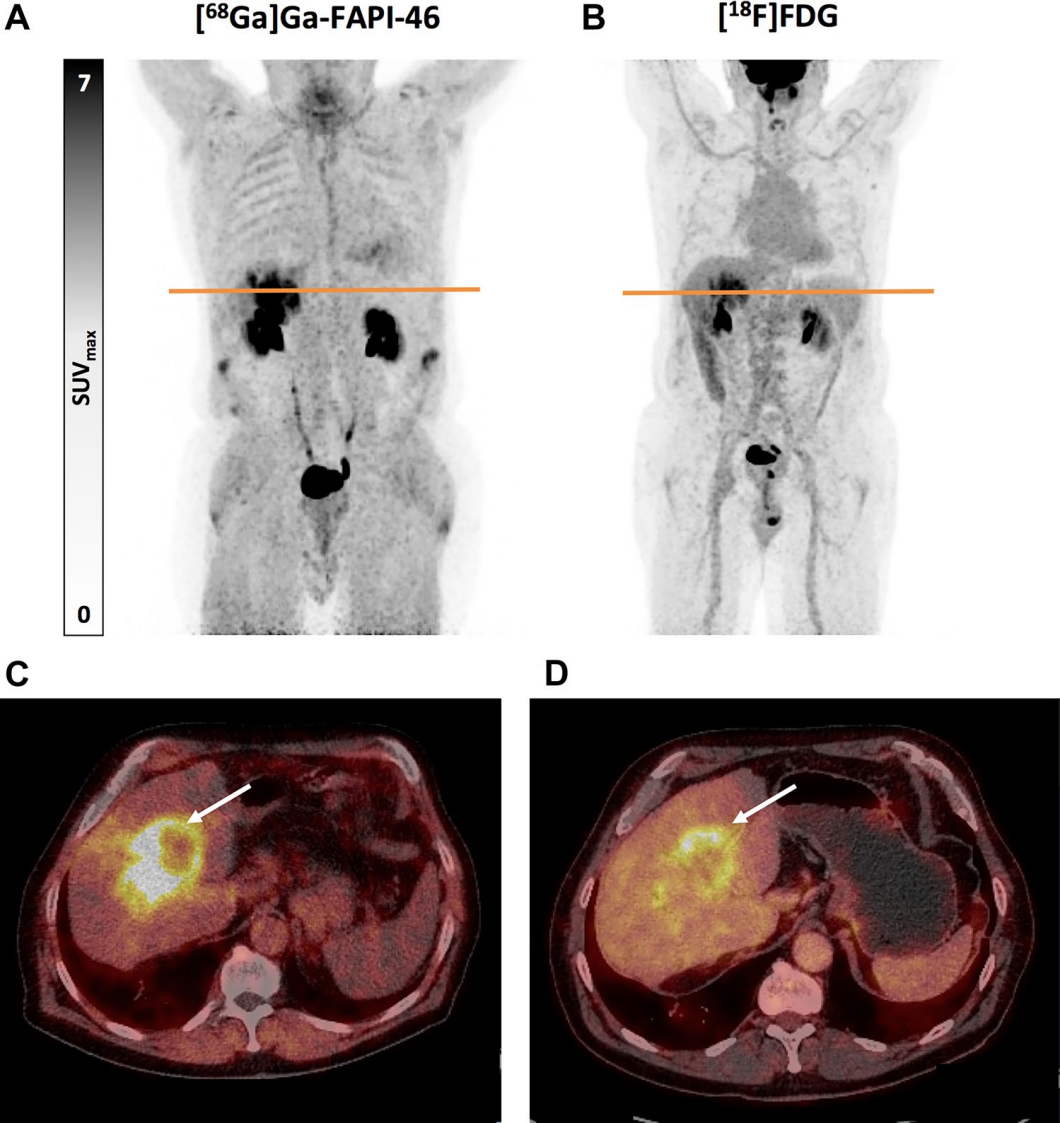
Sixty-seven year old male patient with newly diagnosed intrahepatic cholangiocarcinoma presenting a high tumour uptake on [^68^Ga]Ga-FAPI-46 PET/CT (**A** and **C**) and a moderate tumour uptake on [^18^F]FDG PET/CT (**B** and **D**), with SUVmax 16.6 and 8.6 for [^68^Ga]Ga-FAPI-46 and [^18^F]FDG, respectively. Maximum intensity projection (MIP) PET images (**A** and **B**) and transaxial PET/CT fusion images (**C** and **D**) are presented. Transaxial [^68^Ga]Ga-FAPI-46 PET/CT fusion images (**C**) evidence the high [^68^Ga]Ga-FAPI-46 uptake (white arrow) and the high TBR of the primary tumour with this radiopharmaceutical. In contrast, transaxial [^18^F]FDG PET/CT fusion images (**D**) show a comparatively higher background uptake and, therefore, a lower TBR, which negatively affects the delineation of the primary tumour

Figure 2 presents the case of a newly diagnosed intrahepatic cholangiocarcinoma, showing the differences between [^68^Ga]Ga-FAPI-46 PET/CT and [^18^F]FDG PET/CT.

#### Colorectal cancer

[^18^F]FDG PET/CT plays an important role in the different stages of colorectal cancer, from staging to the suspicion of recurrence [2, 43, 44]. Both radiopharmaceuticals, [^18^F]FDG and FAPI-X have demonstrated a high sensitivity in the evaluation of primary lesions and nodal dissemination (Table 2). It is in terms of specificity where [^68^Ga]Ga-DOTA-FAPI-04 significantly outperformed [^18^F]FDG in these two scenarios (100.0% vs. 85.3% and 100.0% vs. 81.8%, respectively, for primary lesions and nodal dissemination). [^68^Ga]Ga-DOTA-FAPI-04 also showed a better sensitivity than [^18^F]FDG in the detection of peritoneal implants (100% vs. 55%) [45].

### Gynaecological malignancies

[^18^F]FDG-PET/CT is the most used radiopharmaceutical when regarding gynaecological tumours. Among the different malignancies that can affect women we have focused on breast, ovarian and cervical tumours (Table 3).

**Table 3:** Comparison between FAPI-X PET/CT and [^18^F]FDG PET/CT evidence for each clinical application in gynaecological malignancies

	Gynaecological malignancies		
Tumour	Application	FAPI-X	[^18^F]FDG
Breast cancer	T	Higher sensitivity and SUVmax values (17.1 ± 7.9) [47] Higher uptake in invasive lobular carcinoma [50]	Lower sensitivity and SUVmax values (6.3 ± 3.9; p < 0.001) [47] Low uptake in invasive lobular carcinoma [43] FP (i.e. local infection, fibroadenomas, ductal adenomas, mastitis, or fibrocystic changes) and FN (i.e. low tumour volume)
	N	Higher accuracy in LN staging (91.2%) [47, 48]	Lower accuracy in LN staging [47, 48]
	M	Seems to be superior in certain organs with lower FAPI uptake [49]	Limitations in liver, bone and brain metastases [49]
	Response to treatment	Pathologic complete response (pCR) prediction could be assessed by early changes in FAPI PET/CT [51] Changes in post-treatment evaluation [52]	Possibility of underestimating progressive disease after treatment [48]
	Therapy	Allows RPT; favourable results in terms of tolerance and safety [55]	N/A
Ovarian cancer	Uptake	Higher TBR [59] Possibility of distinguishing between benign tumours and borderline tumours, histological subtypes that do not show [^18^F]FDG uptake (i.e. mucinous tumours)	FP (i.e. benign conditions that show increased glycolysis); low sensitivity regarding peritoneal metastases [56]
	N	Higher sensitivity (80.6%) [58]	Lower sensitivity (61.3%, *p* = 0.031) [58]
	M	Higher detection of peritoneal masses (95.0% in the patient-based analysis) [59] Higher accuracy and sensitivity [60]	Lower sensitivity regarding peritoneal metastases (83.3%, *p* = 0.065) [56, 59]
	Reestaging	Higher accuracy for recurrent lesions (97.4%) [62]	Lower accuracy for recurrent lesions (63.9%) [62]
Cervical cancer	T	Similar detection rate for the primary tumour [64]	Similar detection rate for the primary tumour [64]
	N	Higher specificity (100%) [64]	Lower specificity (59.1%, *p* = 0.004) [64]

FAPI-X: FAPI-X PET/CT; [^18^F]FDG: [^18^F]FDG PET/CT; SUVmax: standardized uptake value; TBR: tumour to background ratio; FP: false positive; FN: false negative; pathologic complete response (pCR); N/A: not applicable; NPV: negative predictive value; Uptake: Degree of Radiopharmaceutical Uptake; RPT: radipharmaceutical therapy; T: tumour; N: nodes; M: metastases

#### Breast cancer

Breast cancer is the most common tumour in women, requiring a multidisciplinary approach [46]. In the initial stages, either confined to the breast or presenting regional lymphatic spread, curative treatments are applicable. Thus, accurate staging is crucial. [^18^F]FDG PET/CT may present FP (i.e. local infection, fibroadenomas, ductal adenomas, mastitis or fibrocystic changes) or false negatives (FN) (i.e. low tumour volume, low tumour activity or uptake).

Some studies have investigated the role of FAPI-X PET/CT when compared to [^18^F]FDG PET/CT in staging breast cancer. A prospective study in 24 women (11 invasive lobular, 8 invasive ductal and 5 mucinous subtypes) compared [^68^Ga]FAPI-04 PET/CT and [^18^F]FDG PET/CT, showing higher sensitivity and SUVmax values (mean 17.1 ± 7.9 vs. 6.3 ± 3.9; p < 0.001) when using [^68^Ga]FAPI-04 with notable changes in nodal staging (specially in infraclavicular, supraclavicular and internal mammary) [47]. Another study in 34 women with newly diagnosed breast cancer also showed a better accuracy with [^68^Ga]FAPI-X PET/CT in lymph node staging, especially in N0 cases. The accuracy showed in N staging was 91.2% for [^68^Ga]FAPI-X and 73.5% for [^18^F]FDG [48].

FAPI-X radiopharmaceuticals also seem to be superior to in detecting distant metastases in liver, bone and brain, due to its lower background activity [49]. Combining FAPI-X PET/CT with MRI could also be interesting in primary lesions assessment and lymph node staging [50].

The use of FAPI-X radiopharmaceuticals has also been studied in the prediction of response to neoadjuvant chemotherapy. Neoadjuvant chemotherapy is the treatment of choice in locally advanced breast cancer, also in the inflammatory subtype, in order to allow surgery in this cluster of patients by down-staging primary tumours. A prospective study in 22 patients showed that pathologic complete response (pCR) prediction could be assessed by early changes in [^68^Ga]FAPI-04 PET/CT [51]. [^68^Ga]FAPI-X PET/CT can also modify the management of patients after treatment evaluation by detecting new lesions in the response assessment [52].

As previously cited, some histological subtypes show lower [^18^F]FDG uptake. It is the case of invasive lobular carcinomas when compared to invasive ductal carcinomas [53]. A retrospective study investigated the potential usefulness of [^68^Ga]FAPI-X PET/CT in invasive lobular carcinoma with good performance in terms of evaluation of primary lesions, axillary lymph nodes and distant metastases [54].

Regarding theranostics, [^177^Lu]Lu-DOTAGA.FAPi has been evaluated in metastatic breast cancer patients previously treated with multiple lines with good results in terms of tolerance and safety [55].

#### Ovarian cancer

Ovarian cancer is the third most common gynaecological malignancy and has high rates of mortality. Usually, it is diagnosed in advanced stages due to late symptomatology. Although [^18^F]FDG PET/CT is included in ovarian cancer guidelines, but it has some disadvantages: limitations to distinguish between benign tumours and borderline tumours, histological subtypes that do not show [^18^F]FDG uptake (i.e. mucinous tumours), benign conditions that show increased glycolysis and a low sensitivity regarding peritoneal metastases [56]. Given that it is a challenging tumour, multidisciplinary management is recommended [57].

Some studies have demonstrated higher sensitivity of FAPI-X PET/CT in lymph node evaluation. Chen et al. demonstrated a higher sensitivity with [^68^Ga]Ga-FAPI-04 PET/CT in lymph node detection (80.6% vs. 61.3%; *p* = 0.031) [58]. FAPI-X PET/CT also seems to improve visualisation of peritoneal masses (95.00% vs. 83.33%; *p* = 0.065 in the patient-based analysis) due in part to its higher TBR in comparison to [^18^F]FDG PET/CT [59]. All these findings result in the upstaging of a non-negligible number of patients.

Also, when compared to MRI-diffusion weighted imaging (DWI), FAPI-X PET/CT seems to be superior in the detection of intra-abdominal metastases. A study in 36 patients showed higher sensitivity and accuracy and lower missing rate [60]. The combined use of FAPI-X radiopharmaceuticals with PET/MRI systems can improve management of the patients [61].

FAPI-X PET/CT can also be useful in the management of suspected recurrence. A study in 29 patients with platinum-sensitive recurrent ovarian cancer showed a higher detection of recurrent lesions by using [^68^Ga]-DOTA-FAPI-04 PET/CT compared to [^18^F]FDG PET/CT (accuracy of 97.40% vs. 63.87%) [62].

#### Cervical cancer

[^18^F]FDG PET/CT is included in the cervical cancer guidelines [63]. Regarding FAPI-X, Liu Y et al. compared the diagnostic accuracy of [^68^Ga]Ga-FAPI-04 PET/MRI and [^18^F]FDG PET/CT in patients with T-stage ≤ 2a2. Both radiopharmaceuticals showed a similar detection rate for the primary tumour. The difference was found in the evaluation of lymphatic spread, in which [^68^Ga]Ga-FAPI-04 PET/CT showed a higher specificity (100% vs. 59.1%; *p* = 0.004) [64].

### Thoracic tumours

#### Lung cancer

Lung cancer leads the ranking when referring to lead-cause of deaths by cancer. [^18^F]FDG PET/CT is a well-known tool in the staging and follow-up of the disease and is included in the guidelines [2, 65–67]. However, it is not exempt of FP and FN results. Among the FP findings we find inflammatory or infectious diseases while some subtypes of lung cancer can present a lack of uptake. FAPI-X radiopharmaceuticals uptake depends on lung cancer histopathology with squamous cell carcinomas, adenocarcinomas, large cell neuroendocrine carcinomas and small cell lung cancers showing higher uptake (Table 4) [68].

**Table 4:** Comparison between FAPI-X PET/CT and [^18^F]FDG PET/CT evidence for each clinical application in lung cancer

Thoracic tumours		
Lung		
Application	FAPI-X	[^18^F]FDG
Uptake	Squamous cell carcinomas, adenocarcinomas, large cell neuroendocrine carcinomas and small cell lung cancers showing higher uptake [68]	FP: Inflammatory or infectious diseases while some subtypes of lung cancer can present a lack of uptake [68]
T	Non-inferior [69, 70]	Non-superior [69, 70]
N	Higher SUVmax and TBR and a better specificity [69] Better performance in lymph node metastases (53 and 356 lesions, respectively) [70, 71]	Lower specificity uptake in inflammatory adenopathies [69] Worse performance in lymph node metastases (49 and 320 lesions, respectively) [70, 71]
M	Better performance for pleura (8 and 66, respectively), liver (4), bone (41 and 91, respectively) and brain (23) [70, 71]	Worse performance for pleura (7 and 35, respectively), liver (1), and bone (35 and 91, respectively), and brain (10) [70, 71]

FAPI-X: FAPI-X PET/CT; [^18^F]FDG: [^18^F]FDG PET/CT; SUVmax: standardized uptake value; TBR: tumour to background ratio; Uptake: Degree of Radiopharmaceutical Uptake; T: tumour; N: nodes; M: metastases

[^68^Ga]FAPI PET/CT has already been compared to [^18^F]FDG PET/CT in patients with lung cancer, with variable results. A pilot study performed by Wang R. et al. showed significant differences between [^68^Ga]Ga-FAPI-RGD PET/CT and [^18^F]FDG PET/CT in terms of detection rates. When regarding primary tumour detection, [^68^Ga]Ga-FAPI-RGD PET/CT detected 21/24 lesions while [^18^F]FDG PET/CT detected 16/24. FAPI-X radiopharmaceuticals also demonstrated higher tumour SUVmax and TBR and a better specificity in the evaluation of lymph nodes, as [^18^F]FDG uptake was seen in inflammatory adenopathies [69]. Another study in 28 patients newly diagnosed with non-small cell lung cancer (NSCLC) showed comparable detection of primary lesions but a better performance of [^68^Ga]FAPI-X PET/CT for identifying metastases in lymph nodes (53 vs. 49), pleura (8 vs. 7), liver (4 vs. 1), and bone (41 vs. 35) [70]. The implementation of dual-tracer PET/CT imaging is still to be evaluated in most cancer types, with the potential of providing metabolic information that will maybe help management decisions.

In the scenario of advanced lung cancer, a study in 34 patients, demonstrated a better detection of metastatic lesions in lymph nodes (356 vs. 320), brain (23 vs. 10), bone (109 vs. 91) and pleura (66 vs. 35) in [^68^Ga]FAPI-X PET/CT versus [^18^F]FDG PET/CT. In this study there were no significant differences in the evaluation of primary tumour and detection of suspected metastases in the lungs, liver, and adrenal glands [71].

#### Tumours of the thymus

Thymomas and thymic carcinomas are rare tumours that in general arise in the prevascular (anterior) mediastinum. Thymic carcinomas (or type C thymomas) are usually invasive. Uptake of [^68^Ga]FAPI radiopharmaceuticals has been demonstrated in thymus carcinoma [8]. A prospective head-to-head study by Shen et al. [72] compared the ability of [^68^Ga]Ga-DOTA-FAPI-04 PET/CT and [^18^F]FDG PET/CT in stratifying the malignancy and invasiveness of thymic epithelial tumours. They included 57 patients diagnosed with thymic epithelial tumours that underwent both imaging tests within 1 week. [^18^F]FDG PET/CT can differentiate thymomas from thymic carcinomas, but in this study [^68^Ga]Ga-DOTA-FAPI-04 was superior to [^18^F]FDG PET/CT in this context. In addition, SUVmax in [^68^Ga]Ga-DOTA-FAPI-04 PET/CT increased from low-risk thymomas to thymic carcinoma (median SUVmax ranging from 2.1 to 14.3). Regarding the assessment of the extent of the disease, [^68^Ga]Ga-DOTA-FAPI-04 PET/CT presented better specificity in the detection of lymph node metastases and a better sensitivity in the evaluation of distant metastases [^18^F]FDG PET/CT. They conclude that [^68^Ga]Ga-DOTA-FAPI-04 PET/CT was superior to [^18^F]FDG PET/CT in the evaluation and staging of thymic epithelial tumours [72].

### Endocrine tumours

#### Differentiated thyroid cancer

Differentiated thyroid cancer (DTC) is the most common endocrine neoplasm. The initially diagnosis is based on neck ultrasound with fine-needle aspiration. CT or MRI are performed in case of locally advanced disease or cordal paresis. Its management includes surgery with following radioactive iodine (RAI or [^131^I]) therapy in intermediate or high-risk patients [73].

In DTC, [^18^F]FDG PET/CT is recommended in patients with progressive thyroglobulin (Tg) elevation and a negative RAI scan. Some studies have already demonstrated that the expression of CAFs is related with a more aggressive course and a worse prognosis [73]. In this scenario, the implementation of FAPI-X radiopharmaceutical PET/CT could be interesting. The diagnostic performance of [^18^F]FAPI-42 PET/CT has been already investigated in DTC with biochemical progression with a good rate of detection of local disease, nodal involvement, bone metastases and pleural lesions (mean SUVmax of 3.2 and TBR 4.7). The radiopharmaceutical uptake was not influenced by Tg, TSH or Tg-Antibodies (Tg-Ab) levels. The same study aimed to compare [^18^F]FAPI-42 PET/CT with [^18^F]FDG PET/CT showing a comparable performance between two radiopharmaceuticals [74]. [^68^Ga]Gallium-based FAPI-radiopharmaceuticals have also showed higher detection rates in this scenario [75].

Papillary thyroid cancer is the most common DTC subtype. [^68^Ga]FAPI-04 PET/CT has been studied in patients with biochemical recurrence and an inconclusive [^18^F]FDG PET/CT with higher detection rates and higher SUVmax values with [^68^Ga]FAPI-04 PET/CT. In addition this study showed a relationship between Tg levels and lesion detection (100% accuracy when Tg levels ≥ 301 ng/mL) (Table 5) [76].

**Table 5:** Comparison between FAPI-X PET/CT and [^18^F]FDG PET/CT evidence for each clinical application in head and neck malignancies

	Thyroid cancer and head and neck cancer		
Tumour	Application	FAPI-X	[^18^F]FDG
Thyroid Cancer	Uptake	Lesions with mean global SUVmax of 3.2 and TBR 4.7, not influenced by Tg, TSH or Tg-Ab levels [74]	N/A
	T	Papillary biochemical recurrence and an inconclusive [^18^F]FDG: higher detection rates and higher SUVmax [76]	Papillary biochemical recurrence and an inconclusive [^18^F]FDG: lower detection rates and lower SUVmax [76]
	N	Follicular RAI-resistant: higher detection of lymph node involvement (95.4%, *p* < 0.0001) [77]	Follicular RAI-resistant: lower detection of lymph node involvement (86.6%, *p* < 0.0001) [77]
	M	Follicular RAI-resistant: Higher detection of lung metastases (81.7%); liver metastases (100%, *p* < 0.0001) and brain metastases (100%, *p* < 0.0001); no differences in bone metastases [77] Medullary: Improve the detection of metastases compared to [^68^Ga]Ga-DOTANOC [78]	Follicular RAI-resistant: Lower detection of lung metastases (64.6%); liver metastases (81.3%, *p* < 0.0001) and brain metastases (39% *p* < 0.0001); no differences in bone metastases [77]
	Prognostic	Detection of more aggressive tumours with worse prognosis [73]	N/A
	Therapy	[^177^Lu]LNC1004 promising results in MTD and safety [79]	N/A
Head and Neck Cancer	Uptake	Higher TBR (8.7) [83]	Lower TBR (2.9, *p* < 0.001) [83]
	T	Variable efficiency from similar [84] to better performance [85]. Non-inferior	Variable efficiency from worse performance [84] to similar [85]. Non-superior
	N	Better specificity, avoiding overtreatment [84, 85]	Lower specificity suggesting overtreatment [84, 85]
	Radiation planning	Higher median GTV (57.9 mL) [83]	Lower median GTV (42.5 mL) [83]

FAPI-X: FAPI-X PET/CT; [^18^F]FDG: [^18^F]FDG PET/CT; SUVmax: maximum standardized value; TBR: tumour to background ratio; Tg: thyroglobulin; TSH: thyroid stimulating hormone; GTV: gross target volume; N/A: not applicable; Uptake: Degree of Radiopharmaceutical Uptake; T: tumour; N: nodes; M: metastases; MTD: maximum tolerated dose

#### Follicular thyroid cancer

Follicular thyroid cancer is an uncommon DTC subtype with a greater tendency to metastasize to bone and lung. A retrospective study in 117 patients RAI-resistant showed a higher detection of metastatic lesions by using [^68^Ga]Ga-DOTA.SA.FAPi versus [^18^F]F-FDG PET/CT, specifically in lymph node involvement (95.4% vs. 86.6%; *p* < 0.0001), liver metastases (100% vs. 81.3%; *p* < 0.0001) and brain metastases (100% vs. 39%; *p* < 0.0001). The detection rate of lung metastases was 81.7% in FAPI-X PET/CT and 64.6% with [^18^F]FDG PET/CT. There were no differences in the detection of bone metastases [77].

#### Medullary thyroid cancer

FAPI-X can also be useful in medullary thyroid cancer. [^68^Ga]Ga-DOTA.SA.FAPi PET/CT has been studied in the follow-up, and when compared to [^68^Ga]Ga-DOTANOC PET/CT has proven to improve the detection of metastases [78].

RPT with FAPI-X radiopharmaceuticals has been already proved in radioiodine-refractory thyroid cancer patients. In particular, [^177^Lu]LNC1004 was proved in a cohort of patients with this condition. The primary endpoint was the safety and the maximum tolerated dose (MTD) with promising results in both items [79].

### Head and neck cancer

Head and neck cancer (HNC) are the sixth most common cancer worldwide with increasing rates of incidence and a younger onset in the last years. [^18^F]FDG PET/CT is included in clinical guidelines [2, 80], although it has limitations, such as physiological uptake in cervical structures and FP related to inflammatory tissues.

Epithelial carcinomas are formed in a large percentage by tumour stroma, which makes FAPI-X radiopharmaceuticals an interesting tool in this scenario (Table 5) [81]. The high contrast achieved with FAPI-X radiopharmaceutical PET/CT, due to the low uptake in cervical structures, is interesting in the diagnosis and radiation therapy planning in this cluster of patients [82]. In radiation therapy planning, a study comparing [^18^F]FDG PET/CT and [^68^Ga]Ga-FAPI-46 PET/CT, demonstrated higher median GTV and TBR for FAPI-X radiopharmaceutical (median FAPI-GTV 57.9 mL, FDG-GTV 42.5 mL median FAPI-TBR 8.70 and FDG-TBR 2.94; *p* < 0.001) [83].

In the assessment of primary tumours the results are variable with some studies demonstrating a similar efficiency for primary tumour detection for [^18^F]FDG PET/CT and FAPI-X PET/CT [84] and others showing a better performance for FAPI-X PET/CT [85]. Regarding N staging, FAPI-X has shown better specificity, with a direct impact in the management of these patients by avoiding overtreatment [84, 85].

### Sarcomas

Sarcomas are an uncommon and heterogeneous group of solid tumours and they can involve both soft tissues and bone. They are a challenging group of tumours that require a multidisciplinary approach [86]. FAPI-X radiopharmaceuticals have already been tested in this group of malignancies with promising results (Table 6) [6].

**Table 6:** Comparison between FAPI-X PET/CT and [^18^F]FDG PET/CT evidence for each clinical application in sarcomas

Sarcomas		
Application	FAPI-X	[^18^F]FDG
Degree of Radiopharmaceutical Uptake	Higher uptake in low-grade sarcomas (10.4 ± 8.5, *p* = 0.01) [87]	Lower uptake in low-grade sarcomas (7.0 ± 4.5, *p* = 0.01) [87]
Radiopharmaceutical therapy	Promising results [^90^Y]-FAPI-46 and [^177^Lu]Lu-FAPI-2286 Safety treatment with low adverse effects (mainly laboratory findings) [88, 89]	*N/A*

FAPI-X: FAPI-X PET/CT; [^18^F]FDG: [^18^F]FDG PET/CT; N/A: not applicable; Uptake: Degree of Radiopharmaceutical Uptake; T: tumour; N: nodes; M: metastases

An interesting prospective study by Lanzafame et al. [87] evaluated de diagnostic performance of [^68^Ga]FAPI-46 PET/CT in soft tissue and bone sarcomas, concluding that FAPI-X radiopharmaceutical uptake depends on the different histological subtype of the sarcoma. One interesting result was the notable higher uptake in FAPI-X radiopharmaceutical PET/CT compared to [^18^F]FDG PET/CT in low-grade sarcomas (10.4 ± 8.5 vs. 7.0 ± 4.5; *p* = 0.01).

RPT has also been investigated in sarcomas (Table 7). Fendler et al. suggested some requirements in order to be eligible to receive RPT treatment, the main one being that SUVmax should be > 10 in ≥ 50% of the tumour. [^90^Y]FAPI-46 RPT was studied in a cohort of 21 patients, 16 of them with sarcoma proving it is safe with median PFS 3.4 months (95% CI 1.1–5.7) and median OS 10.0 months (95% CI 4.4–15.5). Adverse effects (n = 51) were mainly related to laboratory findings (88%) [88]. Ferdinandus et al. included 6 sarcoma patients, presenting both metastatic soft-tissue and bone sarcoma, using [^90^Y]-FAPI-46 with promising results in terms of low adverse effects and signs of response [89]. [^177^Lu]Lu-FAPI-2286 has also been studied in metastatic sarcoma patients with promising results [90].

**Table 7:** Adverse events

Authors, year [Ref#]	Tumour	N	Radio-pharmaceutical	Activity (cycles*)	Endpoints	Adverse events (grade 3/4)
Yadav et al. [55]	Breast	19	[^177^Lu]Lu-DOTAGA.FAPi	11–33.3 Gbq (2–6)	P: molecular response (PERCIST criteria). S: OS; PFS; clinical response; safety (CTCAE v 5.0)	No
Fu et al. [79]	Thyroid	12	[^177^Lu]Lu-EB-FAPI ([^177^Lu]Lu-LNC1004)	2.22–4.99 GBq (2)	P: safety; MTD. S: dosimetry; preliminary efficay	Thrombocytopenia
Fendler et al. [88]	Sarcoma, pancreatic, prostate, gastric	21	[^90^Y]-FAPI-46-RPT	3.7–7.4 GBq (1–4)	P: RECIST response; S: PERCIST response; OS; dosimetry; safety	Anemia; thrombocytopenia; altered hepatic markers, abdominal pain
Ferdinandus et al. [89]	Sarcoma, pancreatic	69	[^90^Y]-FAPI-46	0.087–1.335 GBq (1–3)	Safety; dosimetry; response	Anemia; altered hepatic markers; thrombocytopenia
Banihashemian et al. [90]	Sarcoma	8	[^177^Lu]Lu-FAPI-2286	6.66–7.4 GBq (4)	Feasibility; safety; biodistribution; efficacy	No

AE: Adverse events

**Fig. 3:**
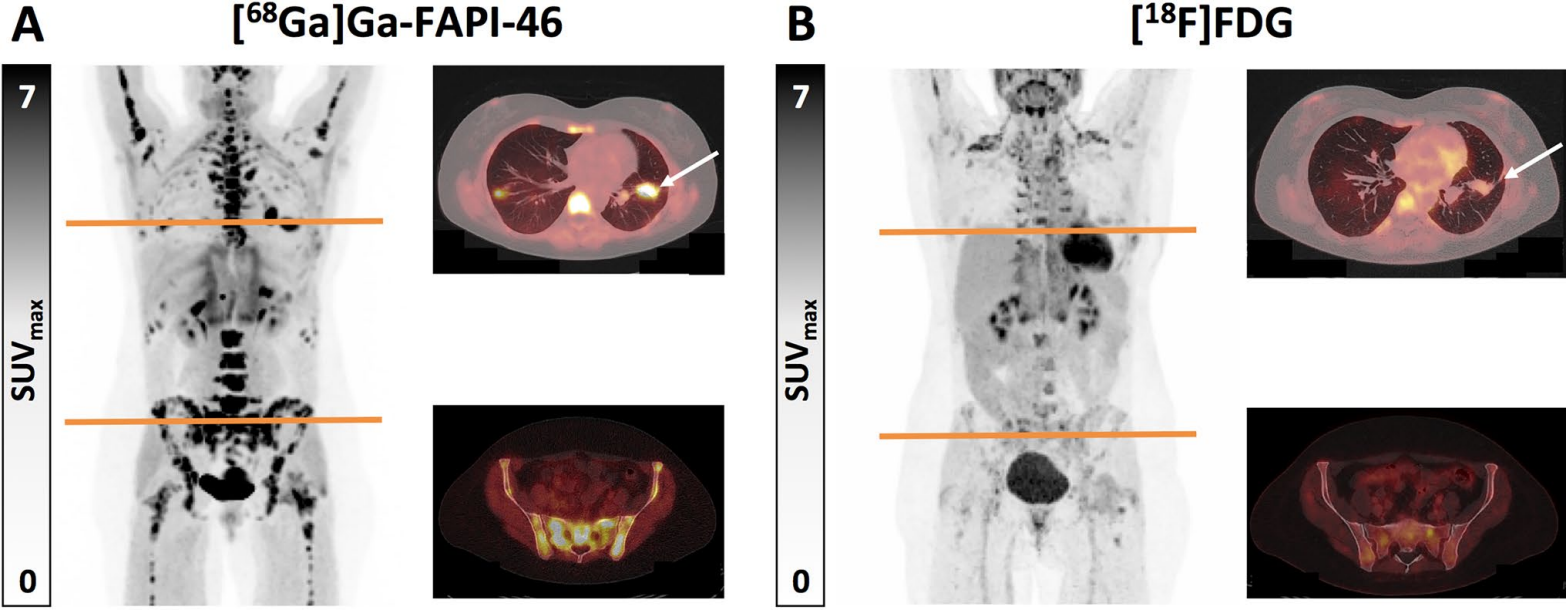
Fifty-two year old female patient who presented a tumour in the right dorsal thigh that had been surgically resected, the pathology study labelling it as a solitary fibrous tumour corresponding to the primary tumour. Re-staging with [^68^Ga]Ga-FAPI-46 PET/CT and [^18^F]FDG PET/CT was performed, detecting pulmonary metastases and diffuse osseous metastases with significantly increased uptake on [^68^Ga]Ga-FAPI-46 PET/CT (A; white arrow, SUVmax 16.7) and low uptake on [^18^F]FDG PET/CT (B; white arrow, SUVmax 3.1)

Figure 3 presents the case of a patient with a solitary fibrous tumour in the thigh. After resection of the primary tumour, restaging with [^68^Ga]Ga-FAPI-46 PET/CT and [^18^F]FDG PET/CT detected lung and bone metastases. The differences between both radiopharmaceuticals are shown.

### Central nervous system tumours

One of the main advantages of FAPI-X radiopharmaceuticals is this scenario is the absence of uptake in brain tissue in normal conditions (Table 8). The study by Liu et al. compared [^68^Ga]Ga-FAPI-04 PET/CT and [^18^F]FDG PET/CT in 25 patients with suspected brain tumours (both gliomas and brain metastases). From a total of 40 lesions discovered by MRI, [^68^Ga]Ga-FAPI-04 PET/CT detected 30, while [^18^F]FDG PET/CT detected 25 (75% vs. 62.5%, *p* = 0.227). TBR was higher in the FAPI studies with a median TBR of 39.9 versus 1.5 in [^18^F]FDG PET/CT (*p* < 0.001). [^68^Ga]Ga-FAPI-04 PET/CT demonstrated uptake in high-grade gliomas with no uptake in low-grade ones. In the case of lymphoma [^68^Ga]Ga-FAPI-04 PET/CT did not overcome [^18^F]FDG [91].

**Table 8:** Comparison between FAPI-X PET/CT and [^18^F]FDG PET/CT evidence for each clinical application in central nervous system tumours

Central nervous system tumours		
Application	FAPI-X	[^18^F]FDG
Uptake	Advantage: low physiological uptake Higher TBR (39.9, *p* < 0.001) [91] High uptake: high-grade gliomas [91]; IDH-wild glioblastomas and high-grade IDH-mutant astrocytomas [92] Low uptake: low grade gliomas and diffuse astrocytomas [92]	Limitation: high physiological uptake Lower TBR (1.5, *p* < 0.001). [91] Lymphoma: non-inferior [91]
T (Tumour)	Better detectability of high-grade gliomas (75%, *p* = 0.227) [91] High PPV can help in unclear MRI lesions [94]	Lower detectability of high-grade gliomas (62.5%, *p* = 0.227) [91]
Radiopharmaceutical therapy	Allows RPT	*N/A*

FAPI: FAPI PET/CT; [^18^F]FDG: [^18^F]FDG PET/CT; TBR: tumour to background ratio; IDH: isocitrate dehydrogenase; PPV: positive predictive value; MRI: magnetic resonance imaging; N/A: not applicable

Based on the histological subtype, gliomas show different FAPI-X radiopharmaceutical uptake. IDH-wild type glioblastomas and high-grade IDH-mutant astrocytomas appear to show a high FAPI-X radiopharmaceutical uptake, while diffuse astrocytomas do not [92]. Gliosarcoma is an aggressive subtype of glioblastoma. It seems to overexpress FAP, which could open a window not only in its diagnosis with FAPI-X radiopharmaceuticals, but also for RPT [93].

Another possible application of FAPI-X radiopharmaceutical imaging in brain lesions could be guided biopsy and treatment planning. A study in 12 glioblastoma patients investigated the role of [^18^F]FAPI PET/CT in pre-radiotherapy assessment. [^18^F]FAPI PET/CT detected 16/23 lesions prior detected in MRI (69.6%). Despite this lower sensitivity, the positive predictive value (PPV) was 100%, which may help in unclear MRI lesions [94].

## Discussion

[^18^F]FDG PET/CT is the most widely used PET/CT radiopharmaceutical in oncology [1–3, 6–8]. Despite its hegemony, it is not exempt from certain constraints and this is the reason for new radiopharmaceutical development. FAPI-X radiopharmaceuticals target the over-expressed FAP in the tumoral stroma which, in some cases may account for as much as 90% of the entire tumour mass. The over-expression of FAP is also related to a worse prognosis. Overall, FAPI-X radiopharmaceuticals seem to be a good partner to [^18^F]FDG PET/CT in some scenarios in which [^18^F]FDG lacks specificity due to inflammatory or infectious uptake or in cases of non-[^18^F]FDG-avid tumours [6, 7]. A recent study compared the diagnostic performance of [^68^Ga]Ga-FAPI-46 PET/CT plus contrast-enhanced CT (CE-CT), [^18^F]FDG PET/CT plus CE-CT, and stand-alone CE-CT in patients with various malignancies. They included 232 patients that underwent the three imaging tests, each within 4 weeks. The detection rates were significantly higher for [^68^Ga]Ga-FAPI-46 PET/CT plus CE-CT than for [^18^F]FDG PET/CT plus CE-CT (*p* < 0.02 for primary lesions and *p* < 0.001 for total, abdominopelvic nodal, liver and other visceral lesions) and CE-CT (*p* < 0.0001 for total, primary, cervicothoracic nodal, abdominopelvic nodal, liver, other visceral, and bone lesions). They concluded that [^68^Ga]Ga-FAPI-46 PET/CT demonstrated a higher tumour detection rate than [^18^F]FDG PET/CT plus CE-CT and CE-CT in a diverse spectrum of malignancies, especially for primary, abdominopelvic nodal, liver, and other visceral lesions [95].

The prognostic value of FAPI-X radiopharmaceuticals has also been analysed in a recent study that compared [^18^F]FDG PET/CT with [^68^Ga]Ga-FAPI-04 PET/CT in patients developing bone metastases due to various cancers. They included 75 patients with 139 bone lesions. [^68^Ga]Ga-FAPI-04 PET/CT detected more bone lesions than [^18^F]FDG PET/CT (*p* = 0.014). The extra lesions observed on [^68^Ga]Ga-FAPI-04 PET/CT were mostly sclerotic bone lesions (*p* = 0.001). While the bone lesion [^68^Ga]Ga-FAPI-04 PET/CT SUVmax affected the OS, the [^18^F]FDG PET/CT SUVmax value did not affect the OS (*p* < 0.001 and *p* = 0.079, respectively). They concluded that [^68^Ga]Ga-FAPI-04 PET/CT detected more bone lesions and higher SUVmax values than [^18^F]FDG PET/CT in various cancers. The prognostic value of the SUVmax value of [^68^Ga]Ga-FAPI-04 PET/CT bone lesions was observed regardless of disease subtype [96].

In certain indications, the use of two or more radiopharmaceuticals may provide relevant information for patient management, supplying detailed information on the biology of the tumours [97]. In addition to its diagnostic capabilities FAPI-X radiopharmaceuticals open a window in RPT with α and β-emitters due to its favourable biodistribution. The main limitation of the studies selected in this non-systematic review is the small cohorts included. Nevertheless, an important percentage of them are prospective studies. However, the methodology of these newly designed studies should aim at harmonising the procedures as much as possible, in order to make results comparable and allow repeatability and reproducibility between different centres [1].

## Conclusions

FAPI-X radiopharmaceuticals present characteristics that can potentially overcome some known diagnostic limitations of [^18^F]FDG. FAPI radiopharmaceuticals present a high target-to-background ratio (TBR) in many solid tumours such as oesophageal cancer, gastric cancer, pancreatic cancer, hepatic cancer, colorectal cancer, breast cancer, ovarian, cervical cancer, and head and neck cancer. Available evidence suggests the high TBR improves sensitivity and specificity compared to [^18^F]FDG, especially for the detection of lymphadenopathies and peritoneal metastases, and may improve patient management and radiation treatment planning. Moreover, it has potential theranostic application. Further evidence is needed to evaluate the precise role of these new radiopharmaceuticals in each clinical indication.

## Data Availability

Not applicable.
